# Using Hormones to Manage Dairy Cow Fertility: The Clinical and Ethical Beliefs of Veterinary Practitioners

**DOI:** 10.1371/journal.pone.0062993

**Published:** 2013-04-26

**Authors:** Helen M. Higgins, Eamonn Ferguson, Robert F. Smith, Martin J. Green

**Affiliations:** 1 Population Health and Welfare Group, School of Veterinary Medicine and Science, University of Nottingham, Sutton Bonington, United Kingdom; 2 Personality, Social Psychology, and Health Research Group, School of Psychology, University of Nottingham, Nottingham, United Kingdom; 3 Division of Livestock Health and Welfare, School of Veterinary Science, University of Liverpool, Neston, United Kingdom; Auburn University, United States of America

## Abstract

In the face of a steady decline in dairy cow fertility over several decades, using hormones to assist reproduction has become common. In the European Union, hormones are prescription-only medicines, giving veterinary practitioners a central role in their deployment. This study explored the clinical and ethical beliefs of practitioners, and provides data on their current prescribing practices. During 2011, 93 practitioners working in England completed a questionnaire (95% response rate). Of the 714 non-organic farms they attended, only 4 farms (0.6%) never used hormones to assist the insemination of lactating dairy cows. Practitioners agreed (>80%) that hormones improve fertility and farm businesses profitability. They also agreed (>80%) that if farmers are able to tackle management issues contributing to poor oestrus expression, then over a five year period these outcomes would both improve, relative to using hormones instead. If management issues are addressed instead of prescribing hormones, practitioners envisaged a less favourable outcome for veterinary practices profitability (p<0.01), but an improvement in genetic selection for fertility (p<0.01) and overall cow welfare (p<0.01). On farms making no efforts to address underlying management problems, long-term routine use at the start of breeding for timing artificial insemination or inducing oestrus was judged “unacceptable” by 69% and 48% of practitioners, respectively. In contrast, practitioners agreed (≥90%) that both these types of use are acceptable, provided a period of time has been allowed to elapse during which the cow is observed for natural oestrus. Issues discussed include: weighing quality versus length of cow life, fiscal factors, legal obligations, and balancing the interests of all stakeholders, including the increasing societal demand for food. This research fosters debate and critical appraisal, contributes to veterinary ethics, and encourages the pro-active development of professional codes of conduct.

## Introduction

Post World War II, scientific and technological advances enabled the industrialization and intensification of agriculture. Concurrently, this has generated a multitude of ethical issues concerning the use of technologies in food production and how farm animals *ought* to be cared for [1], [2]. It is perhaps surprizing, therefore, that veterinary ethics has only recently emerged as an academic discipline; the paucity of literature and lack of any devoted research journal negates an important subject that presents unique challenges, inherently distinct from medical ethics [3]. To start to address this gap in the literature this research concerns the use of a reproductive technology, the prescription of synthetic hormones to manage and improve dairy cow fertility.

There has been a steady decline in the reproductive performance of dairy cows over several decades [4], [5]. Over this time period market forces have driven efficiency savings and lead to genetic selection for production traits, especially higher milk yield. As a result, the specialist dairy Holstein breed is now a substantial component of the UK national herd, managed predominately in a ‘high input high output’ farming system [6]. It is widely accepted that the modern Holstein cow displays less overt signs of oestrus behaviour and for a reduced period of time relative to her lower yielding predecessors [7]. Hence todays farming and veterinary communities are challenged with managing the fertility of an animal that inherently has poorer reproductive performance. Currently in the UK, the annual culling rate for dairy herds is 23% and poor fertility is the commonest reason for culling [8]. In the face of this decline in fertility performance, hormones have been advocated [9] and increasingly been deployed to assist breeding, although to the authors’ knowledge there are no data quantifying the scale of such use currently, nor that has charted this use over time. Hormones, along with all veterinary medicines, are paid for by the farmer.

**Figure 1:**
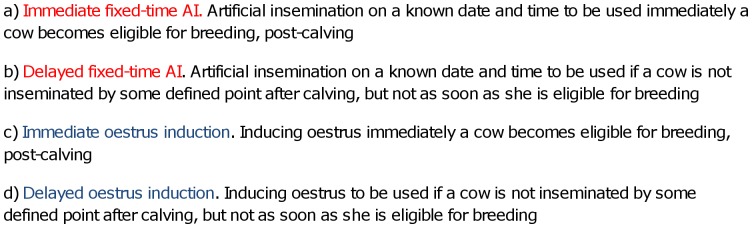
The four main ways to assist breeding in lactating dairy cattle using hormones.

This study concerned three hormones (progesterone, prostaglandin and gonadotrophin releasing hormone), when prescribed to adult lactating dairy cows, *without* reproductive pathology. Two types of use were considered. Firstly, using hormones to induce oestrus - if the farmer knows when to expect to see the cow in oestrus he will observe her more closely and this increases the probability of the cow being served either by the bull or by artificial insemination (AI); this is subsequently referred to as ‘oestrus induction’. Secondly, using hormones over a period of time (often referred to as a synchronization programme) to enable AI on a known date and time; this is subsequently referred to as ‘fixed-time AI’. Oestrus induction requires less hormonal treatments but the farmer must observe the cows several times per day for oestrus (oestrus detection); fixed-time AI involves more hormonal treatments but removes the need for oestrus detection completely. In both cases, hormones can either be used as soon as the cow becomes eligible for breeding after calving, or alternatively, they may only be used if the cow has not been inseminated by the end of a certain period of time, during which she is observed for natural oestrus by the farmer. There are therefore four main ways to assist breeding, as summarised in Figure 1, with (a) involving the greatest quantities of hormones, decreasing in order to (d) with the least. The entire eligible cow population would receive hormones in (a) and (c) but a smaller proportion, depending on the success of natural oestrus detection, in (b) and (d). We focused on the acceptability of use in these four contexts when management problems exist. The acceptability in other scenarios was not explored, such as when unpredictable events occur (e.g. crop failures due to poor weather) which can have a major and unavoidable impact on fertility.

Importantly, in the public eye, the word ‘hormone’ in the context of food production may have negative connotations; historically there has been considerable societal controversy over the prescription of certain hormones to cattle [10]. However ‘hormone’ is a term that classifies a very diverse group of physiological signalling compounds, and the effect and acceptability of use rests entirely on the specific drug and prescribing context. Moreover, in the European Union, all hormones are legally categorised as ‘Prescription Only Medicines Veterinarian’ (POM-V) [11], making them subject to stringent control under legislation contained within Directive 2001/82/EC (as amended) [12]. In the UK this legislation is enforced by the Veterinary Medicines Directorate (VMD), an executive government agency [13], [14]. In particular, manufacturers must prove that any medicine residues in edible tissues are below the statutory ‘maximum residue limits’ and hence safe for consumers health [15], [16]. The VMD monitors on-going safety by continually testing produce for residues [13], [17].

Several stakeholders have vested interests in the debate over the use of this reproductive technology in the context described. This paper explores the issue primarily from the perspective of veterinary practitioners working in private practice in England. As POM-V medicines, administration legally requires prescription from a veterinarian, giving them a central and influential role with respect to how and when these medicines are deployed. Understanding the clinical and ethical beliefs of a range of veterinary practitioners, and any divergence, is important. However to our knowledge, there are no published data on this, or their current prescribing practices. The main aims of this study were therefore: (i) to report the current prescription of hormones to assist breeding by a sample of veterinary practitioners in England, (ii) to explore their clinical beliefs and ethical stance.

## Methods

### Instrument Design

**Figure 2:**
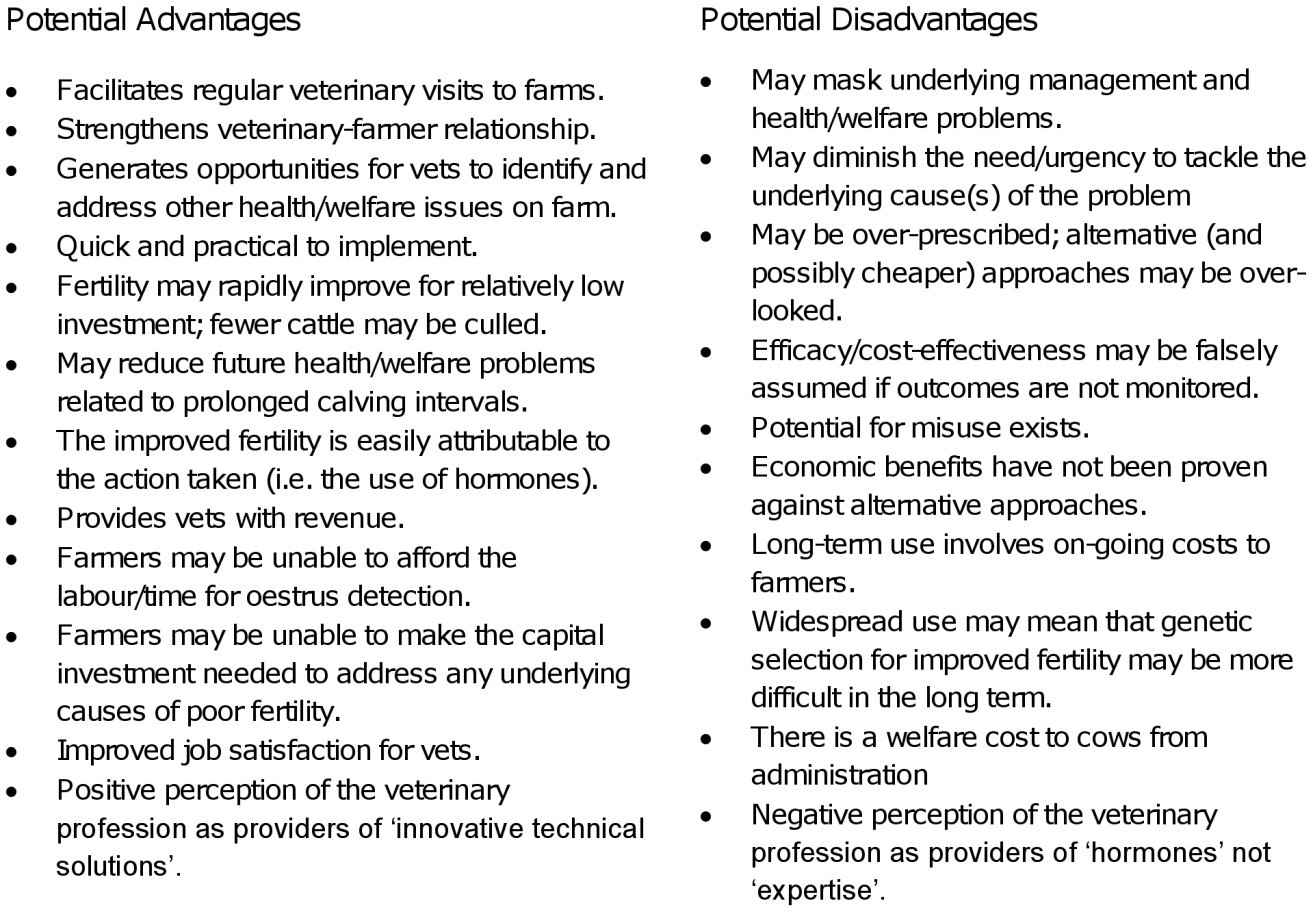
Potential advantages/disadvantages of prescribing hormones from a veterinary perspective. The context relates to the use of hormones to assist breeding in lactating dairy cattle without reproductive pathology. The lists provide a summary of interviews with three veterinary academics and two veterinary practitioners. There is no significance attached to the vertical order of items.

Purposive sampling was used to select two veterinary academics from the University of Nottingham, one veterinary academic from the University of Liverpool and two private veterinary practitioners. Individual semi-structured interviews were conducted by the first author, and the information gathered (see Figure 2) was used to inform and design a questionnaire that was subsequently piloted on two veterinary academics, one psychologist and three veterinary practitioners. The final document comprised a mixture of question formats, and was delivered to a sample of veterinary practitioners (see next section). The questionnaire is available in Appendix S1 and subsequent references to question numbers relate to this Appendix.

### Recruitment of Veterinary Practitioners

Eligible practitioners were those providing healthcare to dairy cattle in England during their normal working hours, and working within a veterinary practice that contained at least one practitioner possessing post-graduate cattle qualifications – specifically, the Royal College of Veterinary Surgeons (RCVS) post-graduate Certificate in Cattle Health and Production or the Diploma in Bovine Reproduction. A two-stage cluster design stratified by geographic location was used. Veterinary practices were selected first, using a ‘without-replacement systematic method’ [19], that involved randomly selecting a starting point and then systematically selecting practices with probability proportional to the number of practitioners they contained. Once 20 practices had consented to take part, five practitioners were then randomly selected from within each practice by using the random number generator function in the software programme ‘R’ version 2.13.1 [18] to pick numerical identifiers; all pracitioners were recuirted in practices that contained less than five eligible practitioners. With this sampling strategy, every individual had approximately the same probability of being selected, irrespective of the size of the practice they worked in. The online database (http://www.rcvs.org.uk/) supplied by RCVS provided a sampling frame of veterinary practices. Practitioners were provided with an inconvenience allowance of £100 per hour (pro-rata). Data were collected from the 8^th^ June to 1^st^ September 2011.

### Data Analysis

The data was initially entered into Excel (Version 2010, Microsoft Corporation). To compare how practitioners’ opinions changed between related categorical questions, two-sided marginal tests of homogeneity were performed [20] (an extension of McNemar’s test for categorical variables) using the software programme SPSS Statistics (Version, 20, IBM); the significance level was <0.05.

Factor analysis was performed using the ‘fa’ function in the ‘psych’ package in the software programme R [18]. The number of factors to extract was based on a combination of: (i) Cattell’s scree plot [21] (ii) eigenvalues greater than 1.0 [22] (iii) interpretability of extracted factors [23] and (iv) chi squared goodness of fit statistic for the maximum likelihood extraction (for a good fit p>0.05) [24]. Sensitivity of the results to the method of analysis was assessed with respect to two different extraction methods (maximum likelihood and principal axes) combined with two different rotations, varimax [25] and promax [26]. A final check on goodness of fit was assessed by the Root Mean Square Error of Approximation (RMSEA); an RMSEA ≤0.06 indicated acceptable fit [27]. Only variables with absolute loading values of ≥0.3 were included in the interpretation of a factor [28]. Variables with little or no variance were excluded from the interpretation. Factor scores (based on all items) for each practitioner were estimated using regression; the distribution of factor scores was assessed to establish majority views across survey questions.

Three logistic regression models were fitted to identify factors associated with responses to three questions in the questionnaire:

- Practitioners reporting concern (yes/no) over the prescription of hormones to assist breeding (question 5).
- Practitioner judgement regarding the acceptability (yes/no) of the long term routine use of immediate fixed-time AI as a substitute for good management i.e. in herds with underlying management problems that are not being addressed (question 16a).
- Practitioner judgement regarding the acceptability (yes/no) of the long term routine use of immediate oestrus induction as a substitute for good management (question 16c).

Questions 16a and 16c carried a ‘don’t know’ option and observations falling in this category were omitted from the analysis. MLwiN software [29] was used and veterinary practice was included as a normally distributed random effect to account for the clustered nature of the data. All models used a logit link function and a penalized quasi-likelihood method for estimation [30]. There were 30 (level 1) covariates available (see Appendix S2). For questions 16a and 16c, factors identified from the factor analysis were also included as covariates. Univariate analysis was conducted and covariates with a P-value of ≤0.05 are reported. Covariates that achieved P≤0.1 were carried forward for model building, and were retained in the final model if they achieved P≤0.05, having adjusted for the other covariates.

The study was approved in full by the Research and Ethics committee, School of Veterinary Medicine and Science, University of Nottingham.

## Results

### Response Rates and Characteristics of Participants

Veterinary practice response rate was 95% (19/20). Non-participation of one practice was due to a failure for all eligible practitioners within it to agree to participate. Another practice was selected and consented, from the same region. These 20 practices contained 95 eligible practitioners, 93 of whom replied, giving a practitioner response rate of 98% (93/95).

Of the 20 practices, 6 were located in the North, 3 in the Midlands, and 11 in the South of England. Table 1 summarises practitioner characteristics.

**Table 1:** Characteristics of veterinary practitioners (n = 94).

Characteristic	Result	
Gender	Male: 59 (63%)	Female: 34 (37%)
Employment status	Partner: 37 (40%)	Assistant: 56 (60%)
Post-graduate cattle qualifications	Yes: 23 (25%)	No: 70 (75%)
Years qualified	Median: 7	Range: 0–37

### Current Prescribing Practices of Veterinary Practitioners in England

Of the 93 respondents, 81 conducted dairy cow fertility work at least once per month on one or more farms; between these practitioners this tallied to 753 farms in total, 39 (5.2%) of which operated under organic regulations that prohibit the use of hormones to assist breeding. Of the 714 non-organic farms, 4 (0.6%) never used hormones to assist breeding, 56 (7.8%) used hormones for immediate fixed-time AI on the majority of cows, 193 (27.0%) used hormones for delayed fixed-time AI on the majority of cows. The remaining 461 farms (64.6%) used hormones to induce oestrus to varying extents, and/or for occasional fixed-time AI.

### Practitioners’ Clinical Beliefs

**Figure 3:**
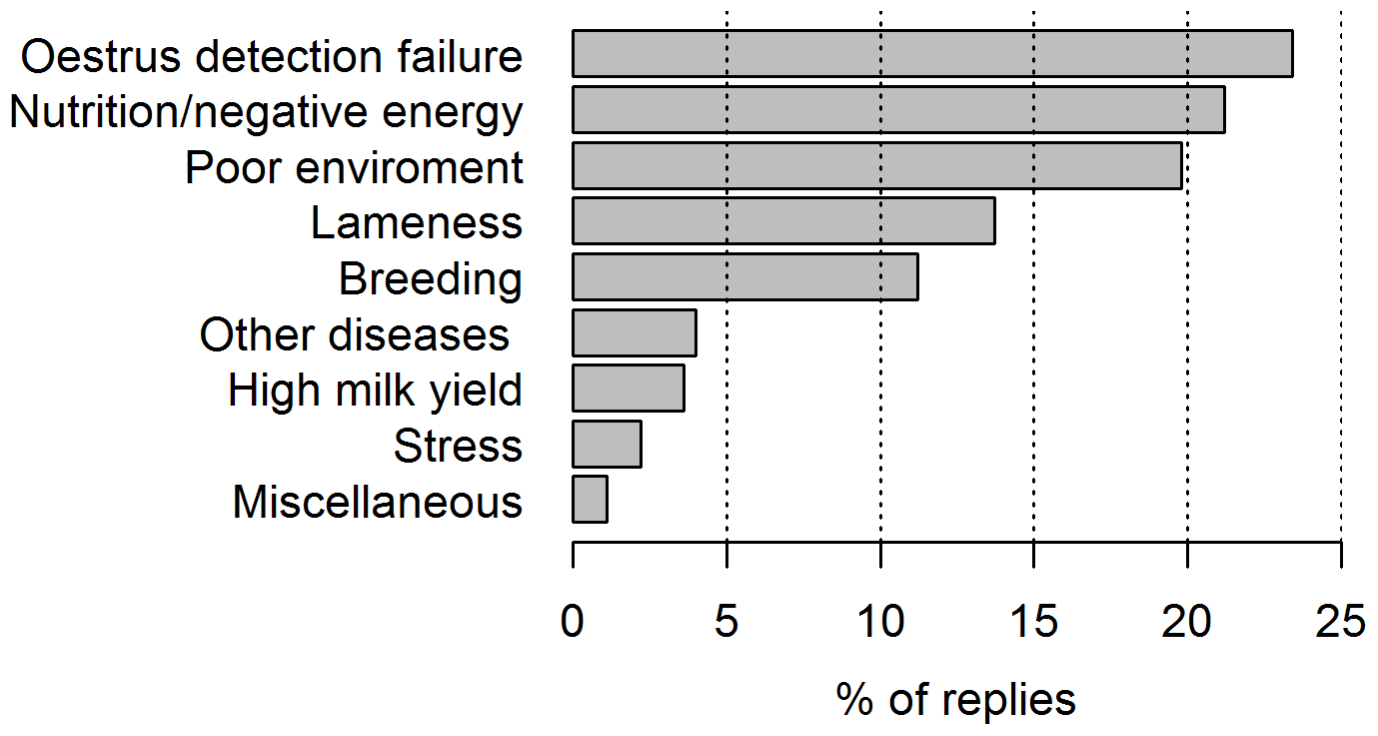
Factors believed to contribute to poor oestrus expression on dairy farms (n = 278 replies). Practitioners’ replies to question 13a were categorized to reflect the answers given, but overlap existed in terms of concepts (e.g. high yield, breeding and lameness are related). The raw data are available in Appendix S3.

A key clinical question was whether prescribing hormones contributes to making any underlying causes of poor oestrus expression, better or worse (Q11). Responses by category were: better 9 (9.8%), no effect 32 (34.7%), worse 33 (35.9%), don’t know 18 (19.6%). With respect to underlying causes, practitioners were asked to list the three most important issues that they believed contributed most often to the problem of poor oestrus expression on dairy farms, see Figure 3.

**Figure 4:**
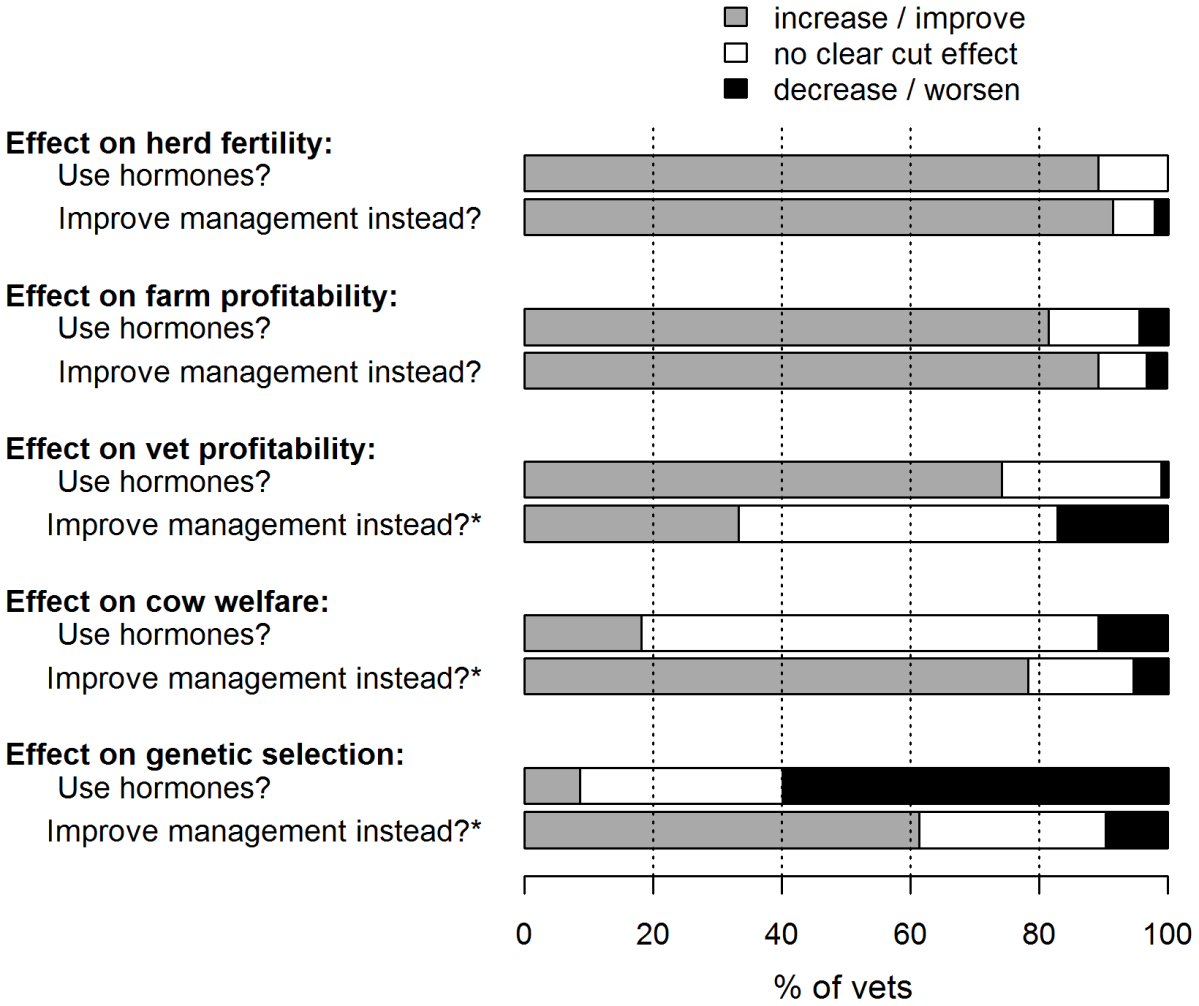
The effect of prescribing hormones versus addressing underlying causes instead over 5 years (n = 93 vets). *denotes a statistically significant change in the distribution of responses between categories, from the outcome if hormones are used to the outcome if management issues are improved instead (p<0.01).

Practitioners also answered a pair of questions, each concerning five key outcomes. The first question asked what effect prescribing hormones would have on each outcome (Q8), and the second question asked what effect tackling the root causes of poor oestrus expression would have after a five year period, if this approach was taken instead of using hormones (13b); the latter was conditional on a crucial assumption, namely that farmers were in a position to make the necessary management changes, including any capital investments required. Responses to the two questions are compared in Figure 4. A two-sided marginal test of homogeneity on this data showed that if underlying management issues are addressed instead of prescribing hormones, practitioners envisaged a less favourable outcome for veterinary practices profitability (p<0.01), but an improvement in genetic selection for fertility (p<0.01) and overall dairy cow welfare (p<0.01).

**Figure 5:**
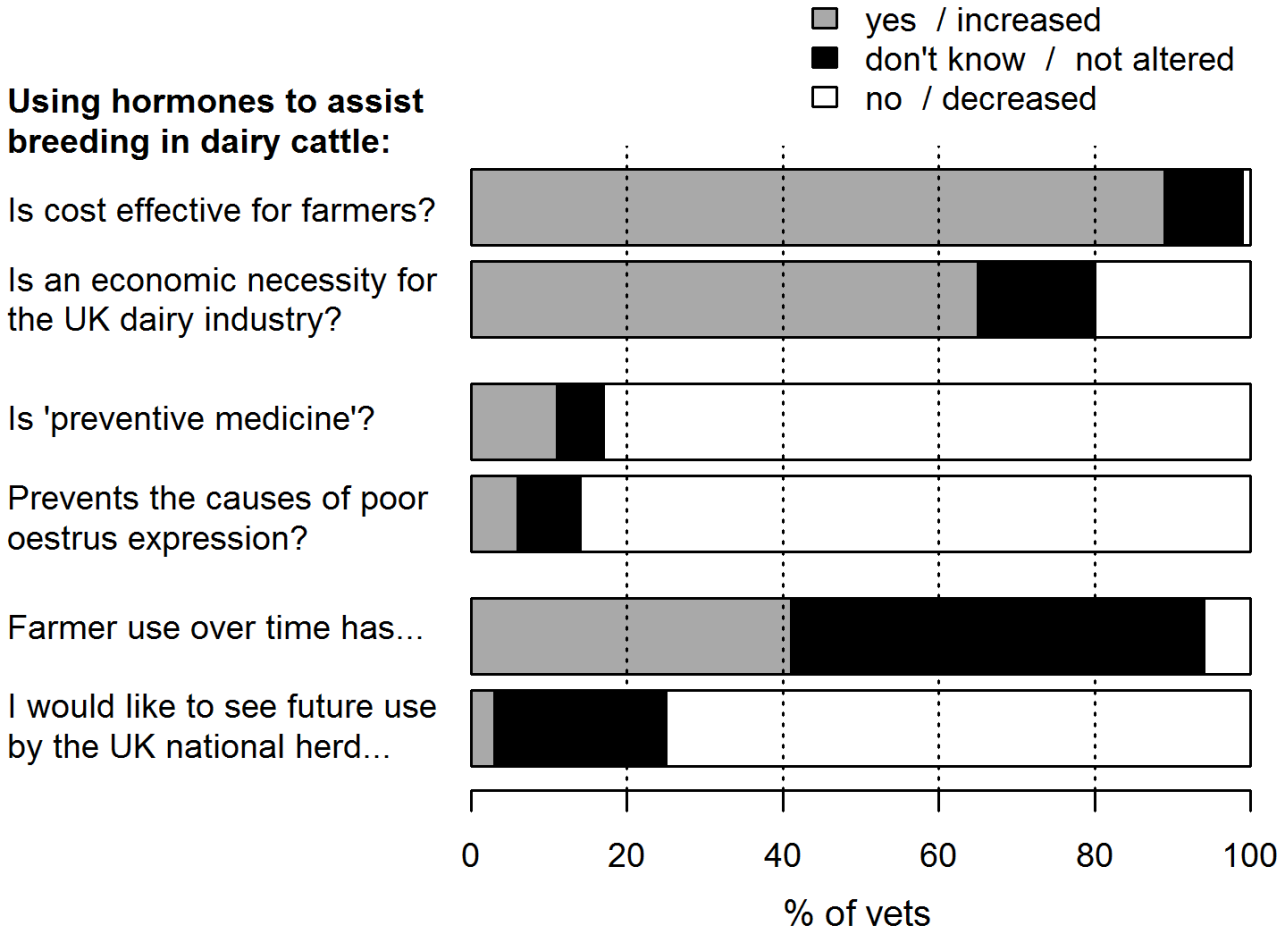
Practitioners’ responses to clinical questions (n = 93). All questions relate to use in lactating dairy cattle, without reproductive pathology. As listed in the figure, these relate to questions 2, 17, 7, 9, 6, and 14 of the questionnaire.

**Figure 6:**
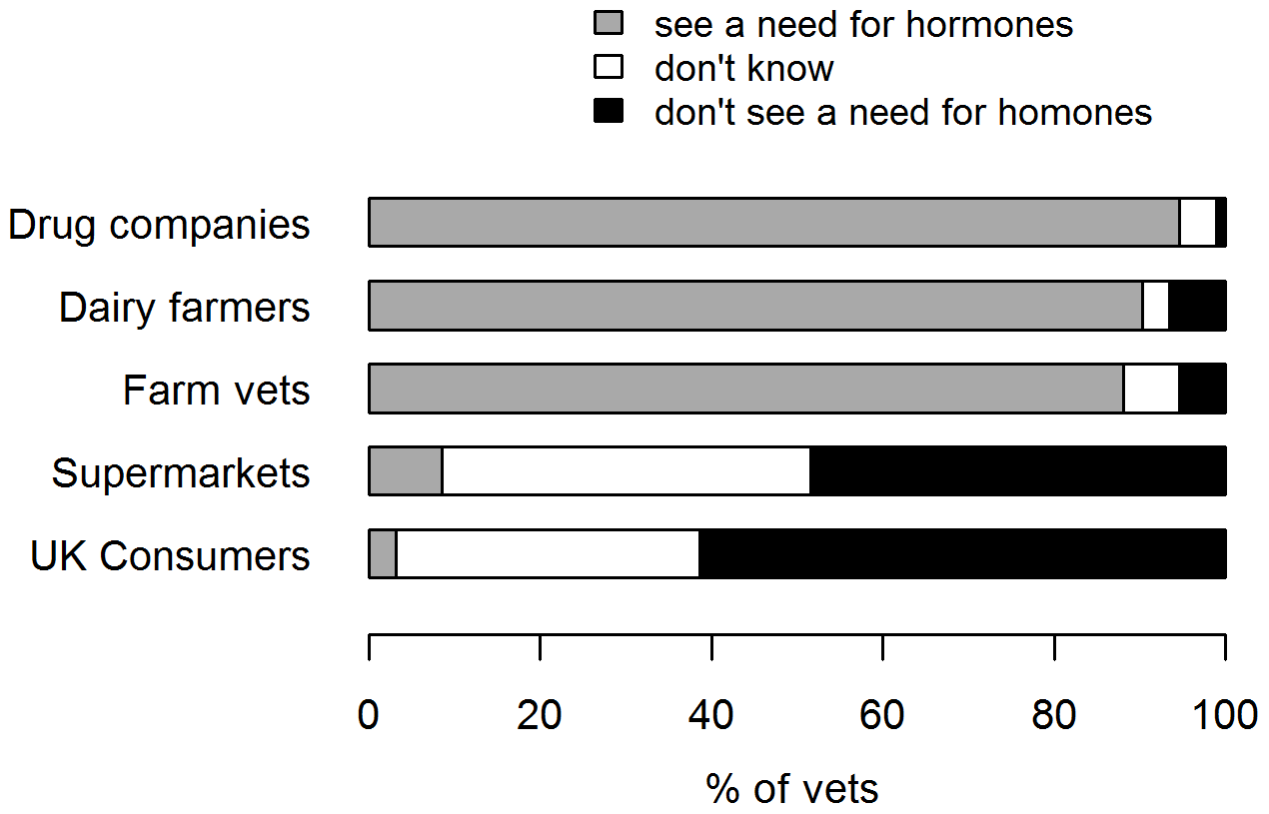
Practitioners’ perceptions of other stakeholders (n = 93). Question 15(a-e) asked practitioners to state whether they believe other stakeholders see a need to prescribe hormones to assist breeding in lactating dairy cattle.

Practitioners’ responses to the remaining clinical questions are given in Figure 5, whilst their perceptions of other stakeholders are illustrated in Figure 6. With regard to decision-making, the main influence over practitioners’ decision to prescribe (Q12) was: veterinarians 46 (51%), farmers 32 (35%), both 13 (14%). Theoretically speaking, if practitioners only had to please themselves and the dairy cow (Q10), then 55 (60%) would use hormones, versus 37 (40%) who would not.

### Practitioners’ Ethical Beliefs

**Figure 7:**
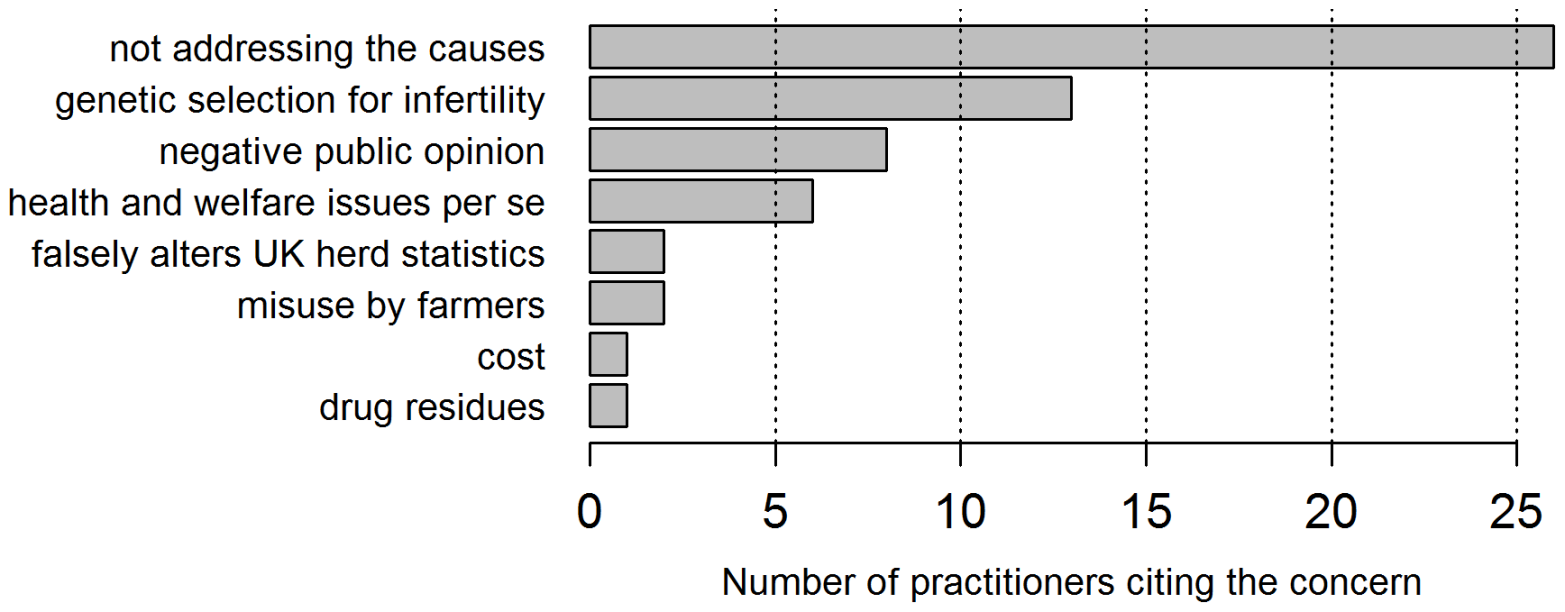
Practitioners’ concerns regarding the use of hormones to assist breeding in lactating dairy cattle (n = 48). Note: some practitioners cited more than one concern.

Practitioners’ responses to the question “Does the use of fertility drugs to get dairy cows served give you any cause for concern?” were divided: 48 (52%) yes, 45 (48%) no. Positive respondents were asked to describe their concerns. Their answers have been categorised in Figure 7, the raw data are available in Appendix S4. The logistic regression results for factors associated with practitioners reporting concern are reported in Table 2.

**Table 2:** Logistic regression results for practitioner characteristics associated with reporting concern over the prescription of hormones to assist breeding.

	Univariable analysis		Multivariable analysis	
Practitioner characteristic+	Odds Ratio(95% CI)	P-value	Odds Ratio(95% CI)	P-value
Currently prescribes hormones for delayedfixed-time AI*?	3.66(1.4–10.3)	0.01		
Attended professional training event on dairycattle fertility within 2 years?	3.89(1.52–9.94)	<0.01	3.72(1.41–9.83)	<0.01
Reports reading the journal ‘UK Vet’?	3.41(1.31–8.85)	0.01		
Number of journals read (odds ratio peradditional journal).	1.54(1.09–2.17)	0.01	1.47(1.04–2.08)	0.03

+ The full list of covariates available is provided in Appendix S2.

* fixed-time artificial insemination to be used if a cow has not been inseminated by some defined point after calving, but not as soon as she is eligible to be bred from.

Practitioners’ ethical beliefs regarding the acceptability in the different prescribing contexts are provided in Table 3. Acceptability was subject in each case to two important conditions: (i) long term routine use, i.e. involving the majority of cows, (ii) prescribing when underlying problems definitely exist that are causing the problem but are *not* being addressed.

**Table 3:** Practitioner acceptability of long-term routine prescription of hormones to assist breeding when management problems are not addressed (questions 16 and 18).

Prescribing context	Number of practitioners		
	Acceptable	Unacceptable	Don’t know
Immediate fixed-time AI	25(31%*)	56(69%)	12
Delayed fixed-time AI	81(90%)	9(10%)	3
Immediate oestrus induction	44(52%)	41(48%)	8
Delayed oestrus induction	87(98%)	2(2%)	4
In general	75(82%)	8(9%)	9

* Percentages relate to the definitive replies.

Factor analysis identified two factors accounting for 30% of the variance. Technical details of this analysis, including the rotated factor matrix are included in Appendix S5. Factor 1 was interpreted as a ‘positive attitude towards the outcomes of prescribing hormones to assist breeding’. Factor 2 was interpreted as a ‘positive attitude towards outcomes if underlying causes of poor oestrus expression are tackled’. Distribution of factor scores suggested that for those variables where there was diversity in opinion, the majority of practitioners tended overall towards both a negative attitude towards the outcomes of using pharmaceutical intervention and a positive attitude towards outcomes that could be achieved if the underlying causes of poor oestrus expression can be addressed.

Logistic regression revealed that only one covariate, years qualified, was positively associated with practitioners judging long term routine use of immediate fixed-time AI acceptable in the face of unaddressed management issues (p = 0.03, OR = 1.05 per extra year, 95% CI 1.04 to 1.11).

Practitioner acceptability of long term routine use of immediate oestrus induction in the face of unaddressed management issues was positively associated with two covariates: (i) the number of farms for which the practitioner was personally currently prescribing hormones for the purpose of any form of oestrus induction (p = 0.02, OR = 1.10 per extra farm, 95% CI 1.02 to 1.24), and (ii) practitioners scores for factor 1 i.e. a positive attitude towards using hormones to assist breeding (p = 0.02, OR = 2.1 per unit increase in score, 95% CI 1.1 to 3.7). It was also negatively associated with one covariate: practitioners score for factor 2 i.e. a positive attitude towards outcomes if underlying causes of poor oestrus expression are tackled (p = 0.02, OR = 0.54 per unit increase in score, 95% CI 0.31 to 0.92). The final multivariate model contained all three covariates with negligible alterations to their parameter estimates or standard errors.

Finally, participants were invited to make additional comments; 25/93 (27%) did so, and some practitioners wrote additional comments throughout the questionnaire (see Appendix S6).

## Discussion

With regard to the acceptability of using hormones to assist breeding in lactating dairy cattle, our results show that even when management problems exist that are causing poor fertility and are not being addressed, the majority of veterinary practitioners judged any type of long term routine use acceptable, provided it was not straight after the start of the breeding period. This may reflect a deontological stance, related to the economic necessity for cows to become pregnant quickly after calving to avoid culling; practitioners’ may consider it wrong to let animals be culled that could be saved, especially given they have a sworn an oath to ‘ensure the health and welfare of animals committed to my care’ [31]. There is also a clinical argument to re-breed cows quickly, since this may reduce the risk of future health problems [32]. Hormonal treatments are quick and easy to implement, however if routine use diminishes the need to tackle root causes, this may have health and welfare implications for the herd. Clinically, there is also the question of whether using hormones contributes towards making any underlying causes of poor oestrus expression better or worse. Veterinary opinion here was ambiguous and divided but not positive, and it is worth noting that time devoted to oestrus detection is also time devoted to disease detection. A utilitarian analysis of ‘do the greatest good for the greatest number’ over a long period may be less supportive of use, although importantly, tackling the root causes may require large capital investment, sustained changes in human behaviour, and take time to resolve; the latter also has implications for sustaining farmer motivation. Two veterinary ethical issues reside here that need advancement. First, how to define and measure a cow’s quality of life, and second how to weigh length versus quality of cow life. In human medicine, the quandary of weighing length versus quality of life has seen ‘quality adjusted life years’ (QALY) used for healthcare resource allocation by organizations such as The National Institute for Health and Clinical Excellence; although QALY is controversial [33] and in need of further research [34]. No equivalent practical decision making tool exists for veterinary practitioners, and the issues involved in developing any such measure are different and arguably even more complex.

Although this survey did not specifically explore how practitioners arrived at their answers with regards to the acceptability of using hormones, some insight can be gained from the additional comments they made. In particular, one practitioner commented “it is [acceptable] in humans”, however there are difficulties with attempting to make reference to seemingly analogous prescribing contexts in the humans. In women the decision to undergo hormonal fertility treatment is a conscious choice, based on knowledge of the advantages and disadvantages of doing so. It is impossible to know if a cow, given the same knowledge (which we cannot impart to them) would make a similar choice to the one that we make for them - and this is assuming that cows can reason. Moreover, the reasons for use and outcomes are very different. Hormones are used to facilitate pregnancy in both fertile and non-fertile animals for reasons related, at least in part, to profitability and human convenience, and non-pregnancy results in culling for human management reasons. In contrast, hormones are only used to facilitate pregnancy in infertile humans, for the sole reason of improving fertility per se, and within a guaranteed non-fatal outcome.

Our results showed that some practitioners did consider it unacceptable to use hormones routinely when management problems are not addressed, especially if conducted at the start of the breeding period. However in reality, the line that separates ‘reasonable assistance to breed’ from ‘a substitute for good management’ may not always be clear-cut. This raises the question of whether veterinary practitioners with a business contract to provide services to a farmer, have a legal obligation to prescribe if requested by a paying client. Legally, as POM-V medicines, the decision to prescribe is the practitioners alone [35]. A farmer who disagreed could attest to a breach of business contact and claim for loss of earnings through a civil court, or in the UK, they could claim professional misconduct to the RCVS. However no precedent has been set and speculating on the outcome is difficult; ordinarily a farmer would terminate the business contract and employ the services of another. By demonstrating the diversity in clinical beliefs and ethical acceptability amongst practitioners, our results suggest that finding someone else to prescribe would not be difficult. Furthermore, veterinarians have professional obligations not only to the animals under their care, but also to farmers and to themselves. Indeed it is postulated that some of the diversity in practitioners’ beliefs related to their empathy towards the various stakeholders which was not directly captured in our survey; this may partly explain why the factor analysis accounted for only one third of the observed variation. In this regard, in the Code of Professional Conduct for Veterinary Surgeons [36], produced by the RCVS, it states: “…veterinary surgeons should balance the professional responsibilities, having regard first to animal welfare.” However, taking an ethical stance as an individual working in private veterinary practice is complicated by the conflicting interests - there is the potential for a substantial loss in revenue. Practitioners have both a need and a right to earn a living, and there are consequences not only for themselves, but also for the support staff that they employ.

Our results suggest that the use of hormones to assist breeding in England is widespread, and the majority of practitioners we surveyed wanted to see future national use decreased. However this represents a challenge for the profession, especially given the conflicting interests described in the previous paragraph. Thus, our results lend support to the *pro-active* development of professional ethical codes of conduct by the RCVS that all veterinarians should abide to. Pursuing a reactive approach is unlikely to be sufficient in the future; the challenge for the 21^st^ century is to provide the rapidly rising world population with a sustainable supply of food, in the context of an increasing demand for animal products, global climate change and declining resources. One practitioner justified their replies in the context of the wider perspective: “all my acceptable answers are making the assumption that the Holstein and its genetics and increased yield and decreased fertility is here to stay to satisfy the need for dairy produce to feed the population.” How to weigh the interests of all stakeholders in the wider context is a crucial question, but currently it is consumers who have the defining influence; in a market-driven playing field it is consumers who collectively, although perhaps unwittingly, drive the efficiency savings and set the economic boundaries within which farmers and veterinary surgeons work. Furthermore, few would argue that it is important for the UK to be able to compete on global market.

With regard to consumers and the public, some practitioners had concerns over negative public perception and the majority believed that UK consumers would not see a need for hormones. It is speculated that these concerns may in part be based on beliefs that the public are not well informed on the issue and that prima facie they would perceive it negatively because it involves hormones, a word that already holds negative connotations for many in the context of food production. However, the actual view of the well informed public is unknown, and needs to be quantified so that the veterinary profession can respond accordingly. In this respect, it is worth noting that in the UK the VMD has a strong track record for ensuring the responsible and safe use of POM-V veterinary medicines and enforcing the legislation contained within the EU Directive [17]. Furthermore there are numerous examples were financial remuneration by doctors has been perceived negatively by the public and adversely affected the level of trust between the public and the medical profession [37]. The inherent financial conflict of interests that inevitably arise from exclusively private veterinary practice will always have the potential to serve as a basis for undermining faith in veterinary expertise. This highlights the importance of studies such as these, which aim in part to inform stakeholders, as well as professional codes of conduct which provide reassurance to the public.

This study has explored some of the issues surrounding the use of one reproductive technology in a given context, but it is not difficult to envisage that veterinary ethical and clinical issues of the type debated here will become more numerous in the future. A key UK government policy for livestock production is ‘sustainable intensification’ [38], and it is proposed that this will be achieved through new scientific and technological advances; thus it is highly probable that, as occurred post World War II, these will bring with them an upsurge in new ethical challenges. Indeed several, such as cloning, are already upon us [3], [4]. There is an urgent need to advance veterinary ethics as a subject and to ensure that it is firmly embedded in undergraduate veterinary curricula. Society has bestowed a considerable responsibility to the veterinary profession for both farm animals and their keepers. In return, society expects us to be acutely aware of the major ethical issues we are part of, to be pro-active and consistent in our approach to tackling them, and to keep the public informed; failure to do so runs the serious risk of loss of autonomy [1], [3].

## Supporting Information

Appendix S1 **The questionnaire.** This file contains the three page questionnaire which was delivered to a sample of veterinary practitioners working in England, UK.
(PDF)

Appendix S2 **Covariate definitions and descriptions.** This file contains details of all the covariates used in the statistical analysis.
(PDF)

Appendix S3 **Factors believed to contribute to poor oestrus expression on dairy farms.** This file contains the free text relies from the 93 practitioners in response to the question: Please list the 3 most important areas that you believe contribute most often to the problem of poor heat expression on dairy farms.
(PDF)

Appendix S4 **Practitioners’ concerns regarding the prescription of hormones to assist breeding in lactating dairy cattle.** This file contains the free text replies detailing the concerns raised by 48 (of 93) practitioners who responded yes to the question: Does the use of fertility drugs to get dairy cows served give you any cause for concern?
(PDF)

Appendix S5 **Factor analysis.** This file contains technical details of the factor analysis.
(PDF)

Appendix S6 **Veterinary practitioners’ additional comments.** This file contains (i) the free text relies detailing the additional comments made by 25 (of the 93) practitioners, in response to the question: If you have other comments about any aspect of the use of fertility drugs to get dairy cows served, or this questionnaire in general, please write them below or overleaf. (ii) additional comments made for any question posed in the questionnaire.
(PDF)
