# Supplementary material for: Using Hormones to Manage Dairy Cow Fertility: The Clinical and Ethical Beliefs of Veterinary Practitioners
Source: PLoS One. 2013 Apr 26;8(4):e62993. doi: 10.1371/journal.pone.0062993 (PMC3637166; doi:10.1371/journal.pone.0062993)
Supplement: Appendix S1 — The questionnaire. This file contains the three page questionnaire which was delivered to a sample of veterinary practitioners working in England, UK. (PDF) [file pone.0062993.s001.pdf]

QUESTIONNAIRE

This questionnaire is about heat expression in dairy cattle. Specifically, it concerns the very common practice of using "**fertility drugs" such as prostaglandins (e.g Estrumate), progesterone (e.g PRIDS), and GnRH (e.g Receptal) to induce oestrus within a predictable, short time period in order to improve the chances of observing a heat OR to allow fixed-time artificial insemination**. Either way, the aim is to get cows served more quickly. Note: this questionnaire is **not** about the use of "fertility drugs" for the purpose of treating uterine disease (e.g. endometritis) or cystic ovarian disease.

We value your opinion because, as a vet in practice, it is you who is faced with the difficult task of managing modern dairy cows. Note: your answers are anonymous and will only be used for independent research purposes for the benefit of the profession. There are no "right" or "wrong" answers to the types of questions we are asking, so please just give us your *honest* opinion. Your help is greatly appreciated, thank-you.

Please note that this entire questionnaire is only concerned with the use of "fertility drugs" in adult lactating dairy cows; this does **include** first lactation heifers but **excludes** maiden heifers.

For each question please circle one answer, or complete the sentence with a number or in word(s) as appropriate:

1) On how many farms do you carry out *adult dairy cow* fertility work on a regular basis, i.e. at least once per month (or for seasonal calving herds, routinely during their breeding season)? ....

Please consider just these dairy farms when answering the next 4 questions (**a-d**):-

a) Of these farms, how many are organic?.......

b) *Excluding* any organic farms, on how many do you ***never*** use ***any*** fertility drugs ***whatsoever*** to get cows served ?........

c) On how many farms do you use *fixed-time AI* on the *majority** of cows *immediately* after the *voluntary waiting period ends*?.....
(* "*majority*" here refers to *more than half* the cows)

d) *Excluding* the farms in your *previous* answer, on how many farms do you use *fixed time AI* on the *majority* of cows that haven't managed to get served by some *specified point* (such as by a certain number of days calved, serves or cycles) but **not** *immediately* after the voluntary waiting period ends?.....

2) Do you believe that the use of fertility drugs to get cows served is *cost-effective* for *most* dairy farmers?
Yes / No / Don't know

3) On dairy farms that have a problem with heat expression but don't already use *any* fertility drugs *whatsoever*, would your approach usually involve advising farmers to start using fertility drugs?
Yes / No / Don't know

4) On dairy farms that have been regularly using fertility drugs on an "ad hoc" basis to get cows served for several years but still have a problem, would your preferred *long-term* aim for these farms be to:

increase / decrease / not alter the amount of fertility drugs the farms use?

5) Does the use of fertility drugs to get dairy cows served give you any cause for concern? Yes / No
If "yes" please describe any concerns in the space below:

6) *Generally speaking*, do you think that *most* dairy farmers who use fertility drugs to get cows served, tend to increase / decrease / not alter the *amount* they use *per cow*, over time?

7) Is the use of fertility drugs to get more cows served, by itself, what you would call "preventive medicine"? Yes / No / Don't know

8) I believe that, *generally speaking*, the use of fertility drugs to get dairy cows served will.......

increase / decrease / have no clear-cut effect on *overall* herd fertility performance

increase / decrease / have no clear-cut effect on farm businesses' profitability

increase / decrease / have no clear-cut effect on veterinary practices' profitability

improve / worsen / have no clear-cut effect on *overall* dairy cow welfare

improve / worsen / have no clear-cut effect on genetic selection for fertility in the *long-term*

9) Do you believe that the use of fertility drugs to get cows served is, by itself, *preventing* the *cause(s)* of poor heat expression? Yes / No / Don't know

10) Imagine a world in which the *only* person you had to *please* was ***yourself*** and no other person (or stakeholder) mattered, *apart* from the *cow*. Would you *use* or *not use* fertility drugs to get cows served?

use / not use

11) In general, do you believe that the use of fertility drugs to get cows served contributes to making any underlying causes(s) of poor heat expression on dairy farms .... better / worse / has no effect / don't know ?

12) Generally speaking, in the past, the main *person* (or group of people) to have influenced my decision over whether or not to use fertility drugs in order to get cows served is........................

13a) Please list the 3 most important areas that you believe contribute most often to the problem of poor heat expression on dairy farms. (*please avoid giving very specific details or largely overlapping subject areas*)

1).. **2)**... **3)**...

13b) Imagine all the dairy farms that are suffering with poor heat expression. The vets and the farmers are going to focus their attention on tackling the areas you have listed above and **all** the farmers are going to be very compliant and implement a large proportion of your advice(!). By the end of a five year period, ***relative*** to what would have been achieved if, instead, ***only*** fertility drugs had been used to get cows served, would you *generally expect* this to

increase / decrease / have no clear-cut effect on *overall* herd fertility performance

increase / decrease / have no clear-cut effect on farm businesses' profitability

increase / decrease / have no clear-cut effect on veterinary practices' profitability

improve / worsen / have no clear-cut effect on *overall* cow welfare

improve / worsen / have no clear-cut effect on genetic selection for fertility in the *long-term*

14) Please consider the *total* amount of fertility drugs *currently* used to get all the dairy cows served in the UK ever year. In *future* years, (assuming the total number of cows stays roughly the same) would *you* prefer to see this total amount... increased / decreased / not altered?

15) For the following list of people/stakeholders, please consider the use of fertility drugs entirely from their perspective and circle whether you believe ***they*** ***see a need*** for fertility drugs to be used to get dairy cows served:

a) The majority of UK dairy farmers see a need / do not see a need / don't know ?

b) The majority of UK consumers see a need / do not see a need / don't know ?

c) The major UK supermarkets see a need / do not see a need / don't know ?

d) Drug companies see a need / do not see a need / don't know ?

e) The majority of UK practising farm vets see a need / do not see a need / don't know ?

16) Consider the four different ways to use fertility drugs listed (**a-d**) below. Please circle for each, if in ***your opinion***, the ***long term routine*** use is **acceptable** (**or not**) in dairy herds which have underlying problems causing poor heat expression but dairy farmers are **not** making any appreciable efforts to address them (NB. Please answer **in principle** and **without** specific reference to the extent to which you personally use these methods on farms)

a) *Fixed-time AI* on the *majority* of cows *immediately* after the voluntary waiting period has ended?

acceptable / unacceptable / don't know?

b) *Fixed-time AI* on the *majority* of cows if they don't get served *by some point* (such as by a certain number of days calved, serves or cycles) but **not** *immediately* after the voluntary waiting period has ended?

acceptable / unacceptable / don't know?

c) *Inducing oestrus* to increase the chance of a heat being observed on the *majority* of cows *immediately* after the voluntary waiting period has ended? acceptable / unacceptable / don't know?

d) *Inducing oestrus* to increase the chance of a heat being observed on the *majority* of cows if they don't get served by some point (such as by a certain number of days calved, serves or cycles) but **not** *immediately* after the voluntary waiting period has ended? acceptable / unacceptable / don't know?

17) Do you think that the use of fertility drugs to get cows served is a *necessity* for the *profitability* of the UK dairy industry? Yes / No / Don't know

18) *Overall*, do you think that the use of fertility drugs in dairy cows for the purpose of getting cows served is ***morally***... acceptable / unacceptable / don't know ?

19) If you have other comments about *any* aspect of the use of fertility drugs to get dairy cows served, or this questionnaire in general, please write them below or overleaf. We welcome and appreciate any comments. *Thank-you.*

....

....

....
