# Supplementary material for: Using Hormones to Manage Dairy Cow Fertility: The Clinical and Ethical Beliefs of Veterinary Practitioners
Source: PLoS One. 2013 Apr 26;8(4):e62993. doi: 10.1371/journal.pone.0062993 (PMC3637166; doi:10.1371/journal.pone.0062993)
Supplement: Appendix S2 — Covariate definitions and descriptions. This file contains details of all the covariates used in the statistical analysis. (PDF) [file pone.0062993.s002.pdf]

Covariate definitions and descriptions

Covariate	Type [levels]
% time dealing with dairy cattle: 0-25, 25-50, 50-75, 75-100	Categorical [4]
Of that time, % time on herd health/prev med? 0-5, 5-10, 10-25, 25-50, >50?	Categorical [5]
Gender: Male (1) Female (0)	Binary
Employed Full time (1) or Part time(0)	Binary
Vet School Attended	Categorical [7]
Years qualified	Discrete
Employment status: Partner, Assistant, Locum	Categorical [3]
Days spent on CPD* within last year: 0, 1-3, 3-5, >5	Categorical[4]
Attended fertility specific CPD event within 2 years? (1=yes, 0=No)	Binary
Awarded the post graduate qualification 'CertCHP' (1=yes, 0=no)	Binary
Awarded the post graduate qualification 'DBR' (1=yes, 0=no)	Binary
Awarded either the CertCHP** or the DBR***? (1=yes, 0=no)	Binary
Awarded another post graduate qualification (excluding 'CertCHP, 'DBR') (1=yes, 0=no)	Binary
Treats equine(1=yes, 0=No)	Binary
Treats 'Small Animals'(1=yes, 0=No)	Binary
Treats 'Other (non-bovine) farm animals'(1=yes, 0=No)	Binary
Self-reported reader of the 'Veterinary Record' journal? (1=yes, 0=No)	Binary
Self-reported reader of the 'Veterinary Times' journal?(1=yes, 0=No)	Binary
Self-reported reader of the journal 'Theriogenology'?(1=yes, 0=No)	Binary
Self-reported reader of the journal 'UK vet'?(1=yes, 0=No)	Binary
Self-reported reader of the journal 'Cattle practice'?(1=yes, 0=No)	Binary
Self-reported reader of 'In Practice'?(1=yes, 0=No)	Binary
Self-reported reader of the 'Journal of Dairy Science'? (1=yes, 0=No)	Binary
Self-reported reader of any other veterinary journal?(1=yes, 0=No)	Binary
Total number of (self-reported) journals read	Discrete
Number of farms vet conducts routine adult dairy cow fertility work on?	Discrete
Vet prescribes 'immediate fixed-time AI on the majority of cows' on one or more farms? (1=yes, 0=No)	Binary
Vet prescribes 'delayed fixed-time AI on the majority of cows' on one or more farms? (1=yes, 0=No)	Binary
Vet prescribes hormones for 'oestrus induction' on one or more farms? (1=yes, 0=No)	Binary
Number of farms vet prescribes hormones for oestrus induction?	Discrete

*continuous professional development i.e. training events
** RCVS Certificate in Cattle Health and Production
*** University of Liverpool Diploma in Bovine Reproduction
