# Supplementary material for: Using Hormones to Manage Dairy Cow Fertility: The Clinical and Ethical Beliefs of Veterinary Practitioners
Source: PLoS One. 2013 Apr 26;8(4):e62993. doi: 10.1371/journal.pone.0062993 (PMC3637166; doi:10.1371/journal.pone.0062993)
Supplement: Appendix S3 — Factors believed to contribute to poor oestrus expression on dairy farms. This file contains the free text relies from the 93 practitioners in response to the question: Please list the 3 most important areas that you believe contribute most often to the problem of poor heat expression on dairy farms. (PDF) [file pone.0062993.s003.pdf]

Veterinary practitioners' free text responses to Question (13a) of the questionnaire:

13a) Please list the 3 most important areas that you believe contribute most often to the problem of poor heat expression on dairy farms. (*please avoid giving very specific details or largely overlapping subject areas*)

1)....................................... **2)** .... **3)**..

There were 278 responses (93 practitioners, but one only gave two answers). Their actual answers are listed under the broad category headings below. Note that these categories are presented to summarise actual veterinary practitioner responses and overlap exists between them in terms of concepts. For example lameness is linked to both poor environment and breeding. In a few cases, clinicians have mentioned two category headings jointly in one answer (e.g. Diets/milk yield) and in this case their answer is listed under the category heading they cite first (in this example, Nutrition).

A. Oestrus detection failure= issues relating to oestrus detection by farmers, including shortage of skilled labour and/or lack of time for observing cows (65/278=23.4%)

1. Heat detection
2. Time
3. Time
4. Poor observation
5. Poor heat detection
6. Time
7. Quality of heat detection
8. Lack of time
9. Shortage of labour
10. Lack of skilled labour and time
11. Poor heat detection
12. Farmers lack of understanding of the need for active heat detection
13. Time/staff availability
14. Poor herdsman/time constraints
15. Time spent observing oestrus
16. Inadequate time to observe oestrus
17. Not enough staff
18. Labour(time)
19. Lack of time spent watching cows
20. Not enough time spent observing cows
21. Poor heat detection

22. Poor detection by farmers
23. Poor heat detection
24. Poor heat detection
25. Heat detection
26. Lack of farmer time
27. Time limitations for farmers to watch their cows
28. Lack of observation
29. Lack of time watching cows
30. Lack of time spent on heat detection
31. Personnel
32. Time
33. Heat detection
34. Time per cow
35. Unobserved
36. Farmer not looking
37. Decreasing farm staff
38. Stockman not enough time to watch for bulling
39. Farm staff skill
40. Labour/observation
41. Oestrus observation
42. Failure to recognise genuine signs of heat
43. Increased herd size relative to labour (more cows per herdsman)
44. Poor observation
45. Poor heat detection
46. Management regimes
47. Quality of heat detection
48. Staff
49. Management
50. Farmer time
51. Poor technique by farmer/knowledge
52. Farmers replacing time spent watching cows with veterinary intervention
53. Lack of time to observe cows
54. Heat detection
55. Lack of skilled farm workers
56. Heat detection
57. Poor heat detection
58. Decreased stockman time
59. Observation periods
60. Farmer not spending sufficient time to observe heat
61. Farm management/stockmanship
62. Presence/absence of testosterone
63. Skill level
64. Stockmanship

65. Poor heat detection

<u>B. Nutrition =Nutritional issues/**N**egative **E**nergy **B**alance (59/278=21.2%)</u>

1. Neg energy balance
2. Neg energy balance
3. Nutrition
4. Nutrition
5. Nutrition
6. NEB
7. Nutrition/BCS/NEB
8. Nutrition
9. Energy deficit
10. Poor Nutrition
11. NEB/BCS
12. Nutrition
13. NEB
14. Nutrition
15. Negative energy balance –weight loss post calving
16. Nutrition (energy deficiency)
17. Poor energy status
18. Nutrition, negative energy state
19. Nutrition
20. Energy levels
21. Feeding
22. Poor diets
23. Diets/milk yield
24. NEB
25. Feeding
26. Nutrition
27. Nutrition
28. NEB
29. Nutrition
30. Nutrition
31. Nutrition
32. NEB
33. NEB
34. Nutrition
35. Nutrition
36. Energy (cow energy)
37. Poor diets nutrition
38. Energy deficient/weight loss
39. Energy balance

40. Poor nutrition
41. Nutrition
42. Nutrition
43. Nutrition
44. NEB
45. Poor Nutrition
46. Nutrition
47. Energy balance
48. Body condition
49. Energy balance
50. NEB
51. NEB
52. Poor transition management
53. Nutrition
54. Poor nutrition
55. Nutrition
56. Nutrition
57. Nutrition/NEB
58. Body condition score
59. Feeding

C. Poor environment=Sub-optimal environment and/or poor cow comfort and/or over-crowding (55/278=19.8%)

1. Housing
2. Poor environment e.g. not enough space, slippy floor, bullying
3. Cow comfort
4. Environment/concrete
5. Poor cow comfort
6. Environment
7. Housing
8. Environment
9. Comfort/space
10. Environment, not enough space for cows to express heat
11. Poor flooring
12. Poor environment
13. Housing/environment
14. Environment
15. Overcrowding
16. Housing
17. Environment
18. Cow space
19. Overcrowding

20. Poor husbandry/flooring
21. Overcrowding
22. High stocking densities
23. Inadequate loafing areas
24. Lack of suitable loafing areas
25. Housing –slippy floors/insufficient space to display behaviour
26. Poor environment
27. Buildings
28. Floor surface
29. Poor areas to show standing heat –floor slippy/lack of space etc
30. Building/yard design
31. Not enough/appropriate space
32. Overcrowding
33. Poor surfaces/grip
34. Housing
35. Lack of loafing areas
36. Accommodation inadequate
37. Not enough loafing space or bullying
38. ‘Cow comfort’/housing
39. Underfoot surfaces/lameness
40. Lack of space
41. Housing
42. Yard surfaces
43. Building design
44. Environment (including stress)
45. Inappropriate environment for expression of heat
46. Stocking density
47. Housing
48. Environment
49. Cow numbers - overcrowding
50. Flooring in yards
51. Buildings
52. Buildings
53. Environment
54. Poor environment
55. Increasing herd size - overcrowding

D. Lameness (38/278=13.6%)

1. Lameness
2. Lameness
3. Lameness
4. Lameness

5. Lameness
6. Lameness
7. Lameness
8. Lameness
9. Lameness
10. Lameness
11. Lameness
12. Lameness
13. Lameness
14. Lameness
15. Lameness
16. Lameness
17. Lameness
18. Lameness
19. Lameness
20. Lameness
21. Lameness/poor surfaces
22. Lameness
23. Lameness
24. Lameness
25. Lameness
26. Lameness
27. Lameness
28. Lameness
29. Lameness
30. Lameness
31. Lameness
32. Lame cows
33. Lameness
34. Lameness-stress
35. Poor locomotion
36. Concurrent disease (e.g. lameness)
37. Lameness
38. Lameness

E. Breeding =Breeding/genetics/Holstein breed (31/278=11.2%)

1. Under fertile cows bred for attributes other than fertility
2. Genetics
3. Genetics/breeding
4. Genetics
5. Genetics
6. Genetics

7. Genetic selection
8. Breeding
9. Poor heat express by Holstein cows
10. Genetics
11. Short heat time in Holsteins
12. Holstein breed
13. Genetics
14. Not strict genetic selection for fertility, allowing cows too many chances to get in calf
15. Breeding
16. Poor expression by Holsteins
17. Breeding
18. Genetics
19. Genetics
20. Genetics
21. Genetics
22. Genetics/breeding
23. Genetics
24. Genetics-push for higher yields seems to be negatively correlated to heat
25. Genetics of current Holsteins
26. Genetics
27. Holstein genetics
28. Genetics
29. Genetics – less hormonal expression of heat
30. Genetics and production
31. Breeding genetics

<u>F. Other diseases =Diseases other than lameness, especially post-partum diseases (11/278=4.0%)</u>

1. Health problems e.g. endometritis, post-calving etc
2. Post partum disease
3. Post partum disease
4. Endomet/pathology of repro tract
5. Cystic ovarian disease
6. Systemic disease
7. Disease
8. Metabolic problems
9. On-going medical problems
10. Metabolic disease
11. Disease

G. High milk yield= High/increased milk yield (10/278=3.6%)

1. High yields
2. Increasing yield
3. High milk yield
4. Milk yield
5. Poor expression due to high yield/nutrition
6. High yielding cows
7. Milk yield/nutrition
8. High yields
9. High yielding
10. Too much pressure for milk production

H. Stress=Stress or metabolic stress (6/278=2.2%)

1. Stress
2. Metabolic stress
3. Metabolic stress
4. Pushing cows too hard
5. Stress
6. Metabolic stress

I. Miscellaneous (3/278=1.1%)

1. Stage of lactation
2. Cow behaviour
3. Insufficient dry period
