# Supplementary material for: Using Hormones to Manage Dairy Cow Fertility: The Clinical and Ethical Beliefs of Veterinary Practitioners
Source: PLoS One. 2013 Apr 26;8(4):e62993. doi: 10.1371/journal.pone.0062993 (PMC3637166; doi:10.1371/journal.pone.0062993)
Supplement: Appendix S4 — Practitioners’ concerns regarding the prescription of hormones to assist breeding in lactating dairy cattle. This file contains the free text replies detailing the concerns raised by 48 (of 93) practitioners who responded yes to the question: Does the use of fertility drugs to get dairy cows served give you any cause for concern? (PDF) [file pone.0062993.s004.pdf]

Veterinary practitioners' reported concerns regarding the prescription of hormones to assist breeding. Question 5:

Does the use of fertility drugs to get dairy cows served give you any cause for concern?

Yes / No

If "yes" please describe any concerns in the space below:

Responses were divided 52% (48/93) yes, 48% (45/93) no. Free text replies from the practitioners who replied yes are listed (in no order) under the broad category headings below. Note: Some practitioners listed more than one concern.

A. Potential substitute for good management, failure to address underlying problems and/or over-reliance (26)

1) "Crutch" for poor management, but essentially an essential control
2) Are we using a sticking plaster rather than sorting out the underlying problem?
3) You haven't addressed the underlying causes adequately
4) Reliance on drug intervention rather than improved management on some units
5) Can mask other issues on farm e.g. health
6) Shouldn't be needed if everything else is corrected e.g. nutrition, management
7) Excuse for poor heat detection
8) If it is a true replacement for husbandry i.e. oestrus observation will not take place at all
9) Better to get management right
10) Only slightly concerned. But do worry/wonder about the underlying reason why the drugs are needed rather than "natural" cyclicity. But not concerned about the use of fertility drugs themselves
11) Reliance on drugs rather than management
12) Not addressing other management issues: ? Poor heat detection? Nutrition etc
13) Underlying factors cause the problem. Use of fertility drugs does not address the problem. Should we be trying to serve cows that are thin/lame etc. so NSB (*not seen bulling*)
14) Use of drugs for "resolving" poor management e.g. poor heat detection.
15) Concern they perceive fertility drugs are easy option and don't put effort into heat detection
16) Use of drugs discourages watching cows for signs of heat and potential reduction in time spent walking around cows (when other things noticed)
17) The use of fertility drugs in non-seen bullers usually reflects husbandry deficiencies which need to be addressed
18) That ignoring a cow in negative energy balance, who is not getting pregnant for a reason e.g. poor management - diet, lameness etc.
19) Often overused to 'make-up' for poor stockmanship or poor environment that means bulling behaviour is not spotted
20) Only if hides poor oestrus observation
21) Improvements can be made in heat detection and transition cow management/nutrition to improve onset of cyclicity and heat expression without the need for drug intervention
22) Should not be used as a replacement for good management i.e. good heat detection etc. tendency to over use
23) It is not a panacea, there needs to be a broad spectrum approach - I wouldn't just use fertility drugs at the expense of other things. Fertility drugs have a place, but nutrition, genetics and herd management should be considered first
24) Over reliance, increase heat detection rate by increase observation and/or aids

25) Often removes clinical judgement/experience
26) No, but I would quantify that by saying that if it got to a ridiculous level then it would concern me and also that my aim is to get cows served to natural oestrus by concentrating on the other management aspects on the farm

B. Possible genetic selection for infertility (13)

1) Genetic selection for infertile cows
2) Are we selecting for cows which don't show heat or at least not selecting against them?
3) Are we breeding cows that are innately sub fertile and becoming reliant on fertility drugs to get in calf?
4) Do we keep on breeding sub-fertile cows?
5) Worry that by getting some cows who struggle to get in calf, in calf by prolonged/heavy drug use we are breeding for infertility in the future
6) Worry that overall cow genetics getting too extreme
7) Breeding future cows which are reliant on fertility drugs to get in calf is not what I how I would like to see dairy fertility going!
8) For future - concerned we are continually breeding replacements from poor fertility animals and are perpetuating problem
9) Possibly reducing fertility overall. Breeding from cows that shouldn't be bred from and therefore producing less fertile offspring; Darwin would pull his hair out!
10) Maybe increasing levels of 'poor fertility' genes by allowing these cows to get pregnant
11) There must be a reason these cows need help of drugs -breeding for poor fertility (due to need or excellent nutrition which they don't often get and bred to produce milk in abnormal quantities)
12) Potentially disguising problems within the breed which would be better bred out
13) If applied standardly on large numbers

C. Negative (or potentially negative) public/consumer opinion (8)

1) Public perception
2) Public perception is v bad
3) Consumer perception
4) Perception outside industry
5) Public opinion (the vegan lobby)
6) Concerned that consumer confidence could be affected by negative spin put on use of fertility drugs. You can see it as positive "helping cows get pregnant" or negative "forcing cows to get pregnant"
7) Potentially from a public perception perspective
8) Public perceived over use of hormones

D. Health/welfare issues per se (6)

1) Cattle health/production long term
2) Cattle welfare
3) Negative impact on welfare e.g. retain cows that have problems e.g. lameness
4) "Forcing" cows to cycle when not fit to do so
5) Cow welfare: so stressed cannot express normal behaviour without intervention
6) Main drivers of the use of such drugs are economic rather than welfare

E. Artificially improves national herd reproduction performance (2)

1) Relying on drugs artificially improves the UK's poor herd reproductive performance
2) Disguises certain fertility issues nationally

F. Misuse by farmers (2)

1) Worry that farmers mis-use/don't correctly follow regimes like ‘ovsync’
2) Handling - giving and leaving farmers drugs the potential to get it wrong and abort something

G. Drug residues (1)

1) Drug residues

H. Cost (1)

1) Cost
