# Supplementary material for: Using Hormones to Manage Dairy Cow Fertility: The Clinical and Ethical Beliefs of Veterinary Practitioners
Source: PLoS One. 2013 Apr 26;8(4):e62993. doi: 10.1371/journal.pone.0062993 (PMC3637166; doi:10.1371/journal.pone.0062993)
Supplement: Appendix S5 — Factor analysis. This file contains technical details of the factor analysis. (PDF) [file pone.0062993.s005.pdf]

Factor analysis.

There were 11 questions posed in the questionnaire that exhibited variance and for which correlation coefficients could be calculated, making them suitable for analysis. Results are reported for the maximum likelihood solution with varimax rotation on the correlation matrix. The results were identical regardless of the method of factor extraction (principal axes or maximum likelihood factor analysis) or the rotation (orthogonal versus oblique). The two factor solution was interpretable and showed acceptable goodness of fit, maximum likelihood chi square p-value=0.23, RMSEA=0.04. Two factors, corresponding to initial eigenvalues of 2.4 and 1.8, accounted for 30% of the variance in total (16% and 14% respectively).

The rotated factor matrix is presented in the table below.

Variable*	Factor	
	1	2
Q4	0.1	-0.05
Q5	-0.5	-0.11
Q6	-0.1	0.04
Q8c	0.02	0.14
Q8d	0.5	0.03
Q8e	0.6	0.04
Q10	0.6	-0.05
Q13c	0.2	-0.1
Q13d	-0.02	0.87
Q13e	-0.06	0.60
Q14	0.6	0.06

*Numbers relate to questions posed in the questionnaire

There were four variables loading positively on Factor 1 (questions 8d, 8e, 10 14), and one variable loaded negatively (question 5). Thus high scores on factor 1 were related to: (i) beliefs that prescribing hormones would increase both genetic selection for fertility and cow welfare, (ii) beliefs that more hormones should be prescribed in the future (iii) practitioners who said they would prescribe if the only stakeholder that mattered (besides themselves) was the cow and (iv) practitioners who did not report concerns over prescribing. Factor 1 was therefore interpreted as a ‘positive attitude towards the outcomes of prescribing hormones to assist breeding’.

There were two variables loading positively on Factor 2: questions 13d and 13e. Thus high scores on factor 2 were related to beliefs that the relative effect of not prescribing hormones would be a positive improvement in overall cow welfare and genetic selection for fertility, given farmers can demonstrably tackle underlying cause of poor oestrus expression. Factor 2 was interpreted as a ‘positive attitude towards outcomes if underlying causes of poor oestrus expression are tackled’. The remaining variables (questions 4,6,8c,13c) had low communalities (< 0.2) and did not load significantly with either factor.

Factor 1 scores were skewed (-0.43, standard error=0.26, skewness ratio 0.83) with the tail of the distribution towards a positive attitude. Factor 2 scores were highly skewed (1.79, standard error=0.26, skewness ratio 3.44) with the tail of the distribution towards a negative attitude.
