# Supplementary material for: Using Hormones to Manage Dairy Cow Fertility: The Clinical and Ethical Beliefs of Veterinary Practitioners
Source: PLoS One. 2013 Apr 26;8(4):e62993. doi: 10.1371/journal.pone.0062993 (PMC3637166; doi:10.1371/journal.pone.0062993)
Supplement: Appendix S6 — Veterinary practitioners’ additional comments. This file contains (i) the free text relies detailing the additional comments made by 25 (of the 93) practitioners, in response to the question: If you have other comments about any aspect of the use of fertility drugs to get dairy cows served, or this questionnaire in general, please write them below or overleaf. (ii) additional comments made for any question posed in the questionnaire. (PDF) [file pone.0062993.s006.pdf]

(i) Veterinary practitioners' free text responses to Question 19 of the questionnaire:

19) If you have other comments about *any* aspect of the use of fertility drugs to get dairy cows served, or this questionnaire in general, please write them below or overleaf. We welcome and appreciate any comments. *Thank-you*.

Twenty five practitioners made comments. Their answers are listed (in no order) below.

1) There is always an underlying reason and this is glossed over
2) Necessity? currently to maintain fertility in some herds as an excuse for poor management
3) As farm size increases and not current milk price, it is inevitable that heat detection will become more difficult for many reasons. Other methods of getting cows in calf will be needed/used. I don't necessarily think this is a good thing!
4) Currently fertility performance is enhanced by use of strategic intervention, having said that some of the best herds use fertility meds very little!
5) As with most vet interventions a combination of client education vet consultation/visits and medication is necessary to maximise fertility
6) Farmers need to take responsibility for ensuring their cows have the best possible chance to express heat in a normal way. If despite all the offerings a cow is not seen to bull then drugs may be used to swing the chance of service back to the farmer
7) Increased/high level of fertility drug use is driven by the industry, both farmers + vets + drug companies. Much peer pressure to use such drugs in order to achieve success in relation to other practice vets
8) I feel the targeted use of fertility drugs is a good thing, but am slightly concerned about the blanket use which makes up for other short falls
9) Good to think about these questions. Profitability-wise for the farmer, fixed time AI as in (16a) is best, but is it in the cow's interest?
10) A dairy cow's survival depends on her fertility. Any help is good!
11) The quandary between getting cows in calf sooner to prevent long calving index -> fat cows -> problems, DA's, ketosis etc.
12) All my "acceptable answers" are making the assumption that the Holstein and its genetics + increased yield and decreased fertility is here to stay to satisfy the need for dairy produce to feed the population
13) The use of fertility drugs to induce oestrus in some cows that haven't shown oestrus by some point is both acceptable and necessary, provided that other possible causes of failure to cycle have been addressed and corrected (e.g. lameness or BCS corrected). Should be minority of cows unless serious problem in herd.
14) Market forces and milk prices are forcing use of all fertility and other drugs
15) Overall morally 'acceptable but we could do more to improve things to reduce their use you have to be realistic in the real world tho!
16) Farmers not made aware enough of potential human health risks of handing these drugs
17) Profitability and efficiency are vital in UK dairy industry to compete globally and supply food at a price that UK consumers will pay. Increasing yields without increasing calving interval is how we need to progress
18) Not the most significant dairy cow welfare issue!
19) The use of the drugs for fixed time AI is not, itself a welfare issue. The problem is the causes of poor fertility in dairy cows
20) Routine fertility visits provide a reason for regular veterinary input on farm. Providing an increase in overall welfare, despite possible welfare concerns over use of fertility drugs; but fertility drug use is preferable to culling
21) Industry become reliant on them as higher yielding cows show less obvious heat

22) I would like to see less fertility drugs needing to be used through increased heat detection as this would help the industry
23) Refer to my point in question 5 - they have a place , but my concern is that they are often used as a front line rather than addressing the other areas because it is easy
24) A necessity
25) I believe we are in a situation where fertility drugs are used too often but that farmers are now looking and breeding/feeding their way back to a more sensible position

(ii) Some practitioners wrote additional comments to questions posed as follows (question numbers relate to the questionnaire, Appendix S1):

6) *Generally speaking*, do you think that *most* dairy farmers who use fertility drugs to get cows served, tend to increase / decrease / not alter the *amount* they use *per cow,* over time?

Of those who replied decrease, there was one extra comment: 'because of cost implications'.

7) Is the use of fertility drugs to get more cows served, by itself, what you would call "preventive medicine"? Yes / No / Don't know

There was one additional comment from someone who replied no: 'a management tool', and one extra comment from someone who replied yes: 'yes, otherwise they could go barren and be culled'.

9) Do you believe that the use of fertility drugs to get cows served is, by <u>itself</u>, *preventing* the *cause(s)* of poor heat expression? Yes / No / Don't know

There was one extra comment from someone who replied yes: 'in some farms'

10) Imagine a world in which the <u>*only*</u> person you had to <u>*please*</u> was ***yourself*** and no other person (or stakeholder) mattered, *apart* from the *cow*. Would you *use* <u>or</u> *not use* fertility drugs to get cows served? use / not use

Of those who stated 'use', one wrote an additional comment: 'but at a lower level i.e. give cows more time'.

11) In general, do you believe that the use of fertility drugs to get cows served contributes to making any underlying causes(s) of poor heat expression on dairy farms .... better / worse / has no effect / don't know ?

Three practitioners who responded 'has no effect' made additional comments: 'but makes up for short falls in husbandry/nutrition/lameness/genetics', 'but means the farmer doesn't have to address it!' and 'the farms who have routine visits and use fertility drugs will often also address other causes of poor heat expression'.

14) Please consider the *total* amount of fertility drugs *currently* used to get all the dairy cows served in the UK ever year. In *future* years, (assuming the total number of cows stays roughly the same) would *you* prefer to see this total amount...   increased   /   decreased   / not altered?

Of those who replied decrease one wrote an extra comment, 'idealistic and probably unrealistic - would prefer natural but doubt this will happen'. Of those who stated a preference for total use not to alter, one also commented 'improved "natural" fertility balancing increased use on currently less proactive farms'.

15) For the following list of people/stakeholders, please consider the use of fertility drugs entirely from their perspective and circle whether you believe ***they* see a need** for fertility drugs to be used to get dairy cows served:

c) The majority of UK consumers…..see a need / do not see a need / don't know ?

Of the respondents who replied 'do no not see a need' one added any extra comment 'because they have no clue it goes on'.

16) Consider the four different ways to use fertility drugs listed (**a-d)** below. Please circle for each, if in ***your opinion***, the ***long term routine*** use is **acceptable** (**or not**) in dairy herds which have underlying problems causing poor heat expression but dairy farmers are **not** making any appreciable efforts to address them (NB. Please answer **in principle** and **without** specific reference to the extent to which you personally use these methods on farms)

a) *Fixed-time AI* on the *majority* of cows *immediately* after the voluntary waiting period has ended?   acceptable / unacceptable / don't know?

Of those who replied 'acceptable' two wrote extra comments: 'but expensive to the industry', and 'but probably will be ineffective'

b) *Fixed-time AI* on the *majority* of cows if they don't get served *by some point* (such as by a certain number of days calved, serves or cycles) but not *immediately* after the voluntary waiting period has ended?   acceptable / unacceptable / don't know?

Of those who replied 'acceptable' two wrote extra comments: 'but expensive to the industry', and 'but probably will be ineffective'

c) *Inducing oestrus* to increase the chance of a heat being observed on the *majority* of cows *immediately* after the voluntary waiting period has ended?   acceptable / unacceptable / don't know?

Of those who replied 'acceptable' two wrote extra comments: 'but expensive to the industry', and 'in some situations'

After question 16, one person wrote: ‘all my “acceptable answers” are making the assumption that the Holstein and its genetics and increased yield and decreased fertility is here to stay to satisfy the need for dairy produce to feed the population’

17) Do you think that the use of fertility drugs to get cows served is a *necessity* for the *profitability* of the UK dairy industry? Yes / No / Don’t know

Three additional comments were made by those answering in the affirmative: ‘at the moment, would prefer it not to be!’, ‘definitely’ and ‘given current margins’.

18) *Overall*, do you think that the use of fertility drugs in dairy cows for the purpose of getting cows served is ***morally***... acceptable / unacceptable / don’t know ?

Three of respondents who replied ‘acceptable’ expanded upon the answer with extra comments, specifically: ‘*in some cases*’, ‘*to a certain level*’ and ‘*it is in humans*’.
